# Highlighting the crucial role of Hangzhou in HIV-1 transmission among men who have sex with men in Zhejiang, China

**DOI:** 10.1038/s41598-017-14108-2

**Published:** 2017-10-24

**Authors:** Jiafeng Zhang, Zhihong Guo, Xiaohong Pan, Wenjun Zhang, Jiezhe Yang, Xiaobei Ding, Yun Xu, Yan Xia, Jianmin Jiang

**Affiliations:** Department of HIV/AIDS & STD control and prevention, Zhejiang Provincial Center for Disease Control and Prevention, Hangzhou, 310051 China

## Abstract

In recent years, the population of men who have sex with men (MSM) constitute a major group for HIV transmission in China. A total of 340 newly reported HIV-infected MSM were recruited proportionally from ten prefectures across Zhejiang province between January and December in 2013. Partial pol gene was amplified and sequenced. Phylogenetic relationship, transmission network and genotypic drug resistance analyses were performed on 311 sequences. HIV-1 subtypes including CRF01_AE (55.9%), CRF07_BC (37.6%), subtype B (1.9%), CRF55_01B (1.3%), CRF68_01B (0.3%), CRF08_BC (0.3%) and URFs (2.6%) were identified. A higher proportion of CRF07_BC and other subtypes existed in the >35 years group, while a higher proportion of CRF01_AE was present in the young group (<35 years). Low prevalence of transmitted drug resistance was found (3.9%, 12/311). Strains with Hangzhou imprints were diffused across the full phylogenetic tree. Moreover, Hangzhou represented the dominant proportion of local HIV transmission (72.0%) and cross-regional transmission (62.4%) based on the provincial transmission network and possessed the largest number of nodes with ≥50 edges, accounting for 50.0% (10/20). The complexity of HIV subtypes and an intertwined network was noticed in MSM in Zhejiang province. Hangzhou likely plays a central regional role in the intra-provincial spread of HIV.

## Introduction

HIV/AIDS continues to stand as one of the most important threats to public health in China. Men who have sex with men (MSM) have accounted for an alarmingly increasing proportion of newly reported HIV/AIDS cases nationally in recent years, rising from 2.5% in 2006 to 25.8% in 2014^1^. A national cross-sectional survey revealed a 4.9% of overall prevalence of HIV infection among the MSM populations^2^. It is clear that MSM have become a major core population of the HIV/AIDS epidemic in China. Therefore, special attention must be given to MSM populations, including the circulating HIV strains among them.

Numerous studies have revealed risk factors of HIV transmission in MSM, including unprotected anal intercourse, multiple sex partners, low rates of condom use, illegal drug use, and a history of other sexually transmitted diseases (STDs)^3–6^. However, studies on HIV subtypes among MSM are inadequate. Previous studies have outlined the profiles of HIV subtypes among Chinese MSM at the national level, demonstrating their dynamics and complexities^7,8^. Considering the widespread disproportion of transmission across the country, deep and detailed studies characterizing localized epidemics are necessary to help implement directional interventions in response to local situations. However, only limited regions, including Beijing^9,10^, Liaoning^11^, Kunming^12^, Anhui^13^ and Shanghai^14^, have reported comprehensive HIV molecular epidemiology studies for local MSM. Relevant studies still should be carried out; these would not only benefit the development of local control measures but also amplify their effects via integration into a cohesive whole-country strategy.

**Figure 1:**
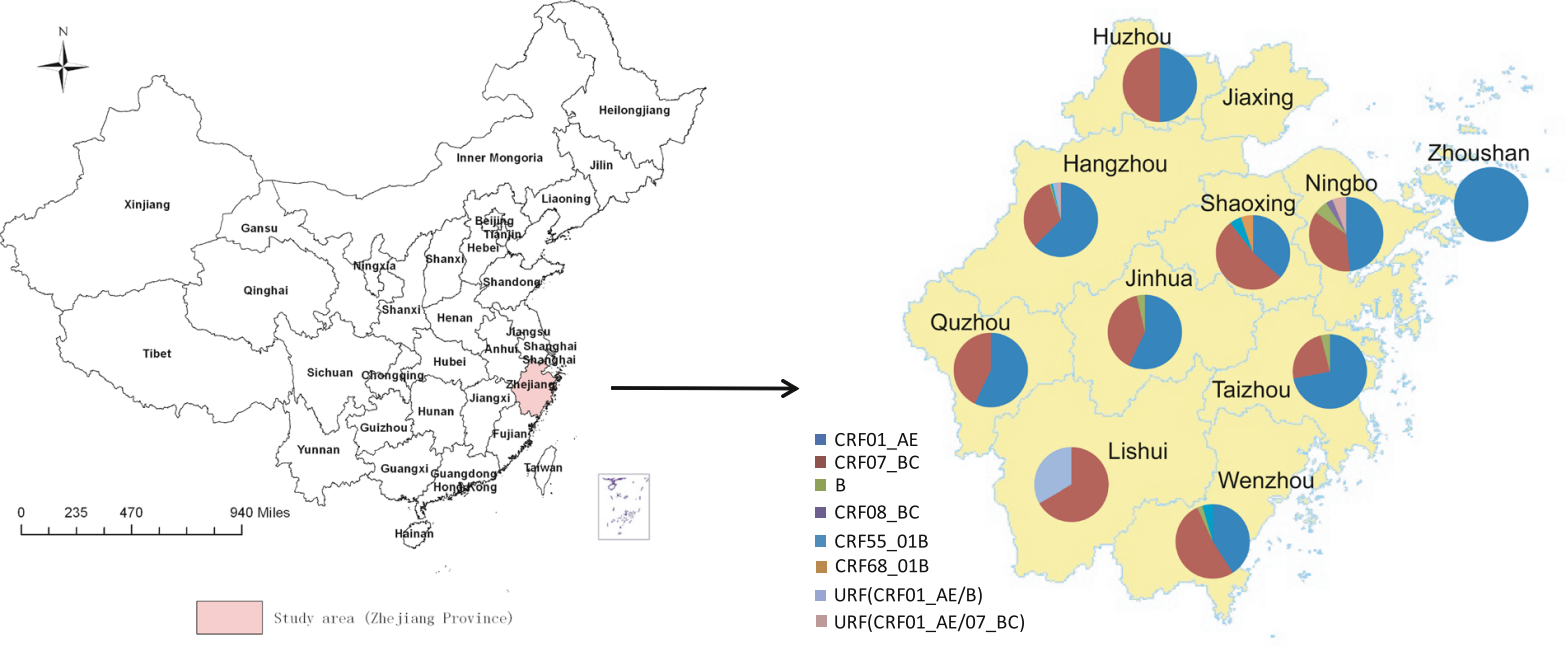
Geographical distribution of HIV-1 genotypes in MSM in the study area. The study area is highlighted on the map of China (left). The distribution of HIV-1 genotypes in each prefecture across Zhejiang province (n = 10), except Jiaxing, is illustrated in the inset on the right. This map was created by ArcGIS software (version 10.1, ESRI Inc.; Redlands, CA, USA) with Homepage of https://www.esri.com/.

Zhejiang, a southeastern coastal province of China (Fig. 1), is one of the most prosperous economic regions in China, attracting a large number of migrants from across the country annually. The first HIV-1 case was identified in 1985 in Zhejiang, and the cumulative number of reported HIV/AIDS cases was 14,703 by the end of 2013, which was the highest number of reported cases in southeastern China. HIV/AIDS cases acquired HIV through homosexual transmission have increased significantly, from 16.1% in 2008 to 37.8% in Zhejiang 2013. The prevalence of HIV infection among the MSM populations in Zhejiang was estimated to be approximately 8% in 2010–2013 as determined through sentinel surveillance^15^ and 13.8% based on a respondent-driven sampling survey^16^, with the highest rate of new infections in focus populations (6.3% in 2012)^17^. Some studies have previously highlighted evidence suggesting great HIV genetic complexity and dynamics^4,6–14,18,19^ and an increased risk of transmitted drug resistance (TDR) among MSM populations^4,10–12,20^. Similarly, our preliminary study revealed a complicated genetic network among MSM in Zhejiang province^21^. However, this study had limitations, including a small sample size and convenience sampling. Therefore, in this study we expanded the number of study subjects by performing stratified proportional sampling based on case reports in each prefecture, attempting to depict the molecular epidemiological characteristics of HIV strains that are prevalent among MSM in Zhejiang province. These findings will help us gain a deeper understanding of the HIV epidemic among MSM in a representative region in southeast China and develop pointed measures to curb the rapid spread of HIV-1 in this population.

## Results

### Demographic Characteristics of the Study Patients

A total of 340 eligible subjects were enrolled in the study across Zhejiang province. The mean age was 31.9 ± 1.0 years old (range, 14 to 68 years). The majority of the subjects were single (n = 218 [64.1%]), of Han ethnicity (n = 328 [96.5%]), had achieved beyond compulsory education (n = 137 [40.3%]) and permanently resided in Zhejiang province (n = 177 [52.1%]). The subjects were involved in various occupations: commercial service workers accounted for 30.9%, followed by workers and peasants (28.8%) and cadres, students, teachers and retirees (12.6%) (see Table 1). Among the means of discovering their HIV infection status, voluntary counseling and testing (VCT) constituted the greatest proportion.

**Table 1:** Demographic Characteristics of Studied Subjects.

Categories		Subjects	HIV subtypes			χ2	*P* value
			CRF01_AE	CRF07_BC	Others		
Age	≤25	101 (29.7)	58	34	2	15.860	0.003
	26–35	138 (40.6)	78	43	6		
	>35	101 (29.7)	38	40	12		
Nationality	Han	328 (96.5)	168	113	19	0.611	0.779^a^
	Minority group	12 (3.5)	6	4	1		
Household registered	Zhejiang	177 (52.1)	86	64	10	0.797	0.671
	Other provinces	163 (47.9)	88	53	10		
Marital status	Single	218 (64.1)	117	77	7	13.384	0.008
	Married	62 (18.2)	24	20	10		
	Divorced/widowed	60 (17.6)	33	20	3		
Education status	Primary school/illiterate	34 (10.0)	14	13	3	4.149	0.657
	Junior high school	103 (30.3)	50	37	7		
	Senior high school	87 (25.6)	47	25	6		
	College or above	116 (34.1)	63	42	4		
Present address	Hangzhou	143 (42.1)	87	45	7	4.518	0.101
	Other regions	197 (57.9)	87	72	13		
Occupation	Commercial service workers	105 (30.9)	58	30	8	12.866	0.195^a^
	Workers and peasants	98 (28.8)	48	34	5		
	Cadres, doctors, teachers, students and retirees	43 (12.6)	21	18	3		
	Domestic workers and unemployed	35 (10.3)	22	9	0		
	Service workers in public places	22 (6.5)	7	14	1		
	Other and unknown	37 (10.9)	18	12	3		

^a^Fisher’s exact test.

### HIV-1 Subtype distribution

Twenty-nine out of 340 samples failed to amplify, while 311 (91.5%) were successfully amplified, sequenced, and subjected to genotyping. Neighbor-joining phylogenetic tree analysis performed using MEGA 6.0 revealed that CRF01_AE was the dominant subtype (55.9%, 174/311), followed by CRF07_BC (37.6%, 117/311), subtype B (1.9%, 6/311), CRF55_01B (1.3%, 4/311), CRF68_01B (0.3%, 1/311), CRF08_BC (0.3%, 1/311), URF(CRF01_AE/B) (1.3%, 4/311) and URF(CRF01_AE /07_BC) (1.3%, 4/311). The geographic distributions of HIV-1 genotypes among MSM were summarized in Fig. 1.

There were no statistically significant differences in terms of the subtype distributions among the involved prefectures (*P* = 0.079). CRF01_AE and CRF07_BC were the two most dominant strains circulating in each prefecture, with total proportions ranging from 66.7% to 100.0%. Novel CRFs (such as CRF55_01B and CRF68_01B) and URFs were found sporadically in many locations. Spatial distribution of the CRF55_01B strains involved Hangzhou (n = 1), Wenzhou (n = 2) and Shaoxing (n = 1). CRF68_01B (SX130239) was first identified in Shaoxing, which was the second case reported in Zhejiang province. URF(CRF01_AE/B) was distributed in Hangzhou (n = 3) and Lishui (n = 1), while URF (CRF01_AE/07_BC) was found in Hangzhou (n = 2) and Ningbo (n = 2).

HIV subtype distribution exhibited statistically significant differences in age and marital status, with χ2 = 15.860 (*P* = 0.003) and χ2 = 13.384 (*P* = 0.008), respectively. However, no statistically significant differences were observed for categorical variables, including nationality, residence, education status and occupation (*P* > 0.05). Pairwise comparisons in the age subgroup revealed a significant difference between the ≤25-year group and the >35-year group (χ2 = 11.715, *P* = 0.003), as well as the 26–35-year group and the >35-year group (χ2 = 9.880, *P* = 0.007). A higher proportion of CRF07_BC and other subtypes was present in the >35-year group compared to the younger group (<35 years), while the latter had a relatively higher proportion of CRF01_AE. Similarly, a significant difference was found between the single group and the married group (χ2 = 15.911, *P* < 0.001). A higher proportion of CRF01_AE was distributed in the single group, while a notably higher proportion of other subtypes was found in the married group. All study participants included in this analysis are outlined in Table 1.

### Characteristics of Novel CRFs/URFs

As shown in Table 2, the subjects infected with novel CRFs/URFs demonstrated a large age span, ranging from 19 to 56 years old, but 8 cases (61.5%) were concentrated in the >35-year group. Nearly half (46.2%) of subjects residing in Hangzhou were confirmed as HIV-positive and reported HIV infection locally. Eight cases (61.5%) were migrants from other provinces, including Hunan, Shandong, Jiangxi, Yunnan, Jiangsu, Shaanxi and Shandong. The occupations of these cases were widely dispersed, involving at least eight different careers, of which commercial service workers accounted for the largest proportion. A total of 61.5% (8/13) of patients infected with novel CRFs/URFs were married or divorced/widowed, while the others were single. Additionally, 69.2% (9/13) had CD4^+^ T lymphocytes counts less than 500 cells/µl.

**Table 2:** Characteristics of Patients with Novel Subtypes and URFs.

Sample ID	Age	Diagnosis date	CD4 count	Location of reported cases	Household registered	Present address	Occupation	Marital status	HIV Subtypes
HZ130207	27	2013/6/20	188	Hangzhou	Hangzhou	Hangzhou	Teacher	Single	URF(CRF01_AE/B)
HZ130739	22	2013/6/27	311	Hangzhou	Hunan	Hangzhou	Student	Single	URF(CRF01_AE/B)
HZ130991	53	2013/7/4	168	Hangzhou	Hangzhou	Hangzhou	Peasant	Married	CRF55_01B
HZ130947	45	2013/5/3	28	Hangzhou	Hangzhou	Hangzhou	Commercial service worker	Married	URF(CRF01_AE/07_BC)
HZ130198	29	2013/5/15	533	Hangzhou	Shandong	Hangzhou	Other	Single	URF(CRF01_AE/07_BC)
HZ130743	56	2013/7/4	566	Hangzhou	Hangzhou	Hangzhou	Worker in food service industry	Married	URF(CRF01_AE/B)
WZ130854	37	2013/5/30	527	Wenzhou	Jiangxi	Wenzhou	Worker	Divorced/widowed	CRF55_01B
WZ130115	29	2013/6/21	515	Wenzhou	Yunnan	Wenzhou	Commercial service worker	Single	CRF55_01B
NB132624	49	2013/12/5	186	Ningbo	Jiangsu	Ningbo	Peasant	Married	URF(CRF01_AE/07_BC)
NB130937	48	2013/4/23	328	Ningbo	Shaanxi	Ningbo	Commercial service worker	Married	URF(CRF01_AE/07_BC)
SX130239	51	2013/3/26	279	Shaoxing	Shaoxing	Shaoxing	Commercial service worker	Divorced/widowed	CRF68_01B
SX130580	19	2013/7/2	279	Shaoxing	Hunan	Shaoxing	Commercial service worker	Single	CRF55_01B
LS130100	45	2013/3/25	210	Guangzhou	Shandong	Lishui	Unknown	Married	URF(CRF01_AE/B)

**Figure 2:**
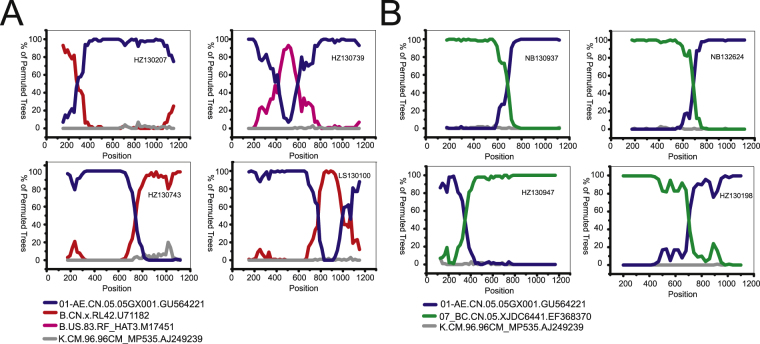
Bootscanning analysis of the recombination structures for newly identified URFs. Recombination patterns of CRF01_AE/B and CRF01_AE/CRF07_BC are shown in Figure A and Figure B, respectively. Unless otherwise specified, the general conditions for bootscanning analysis were as follows: Window: 300 bp, Step: 20 bp; GapStrip: on, Reps: 100, Kimura (2-parameter), T/t: 2.0, Neighbor-joining. Some analyses were performed after slight adjustments to the Window and Step sizes. The exception sequences include NB130937 (Window: 350 bp), NB132624 (Window: 350 bp), HZ130947 (Window: 250 bp) and HZ130198 (Window: 400 bp, Step: 30 bp). The reference sequences, including CRF01_AE, B, B’, CRF07_BC and K (outlier), are shown at the bottom left of the figure.

There were eight sequences that did not match known CRFs and thus were classified as URFs (2.6%), of which four cases (HZ130207, HZ130739, HZ130743 and LS130100) were determined to be CRF01_AE/B, and the remaining four cases (NB130937, NB132624, HZ130198 and HZ130947) were CRF01_AE/CRF07_BC. The recombination patterns were depicted further through informative site analysis using Simplot v3.5.1 (Fig. 2). The isolate HZ130207 displayed a recombination structure with subtype B and CRF01_AE identified at positions 2253–2527 and 2528–3460, respectively. The isolate HZ130739 showed a recombination structure with CRF01_AE segments identified at positions 2253–2664 and 2847–3448 and B segments at position 2665–2846. The isolate HZ130743 exhibited a mosaic recombination pattern with CRF01_AE segments determined at position 2253–2995 and subtype B segments at position 2996–3448. Regarding isolate LS130100, mosaic construction was detected with CRF01_AE segments determined at positions 2253–3025 and 3248–3432 and subtype B segments at position 3026–3247. It was intriguing that the strains NB130937, NB132624 and HZ130198 demonstrated a high degree of similarity in terms of their recombination patterns. For example, NB130937 exhibited a recombination structure with CRF07_BC segments identified at position 2253–2934 and CRF01_AE segments at position 2935–3463. The isolate HZ130947 possessed a unique mosaic pattern with CRF01_AE segments detected at position 2253–2593 and CRF07_BC segments at position 2594–3455.

By performing BLAST with worldwide HIV sequences, we determined that 4 cases of CRF55_01B in this study demonstrated a close relationship with strains circulating in Shenzhen and Guangzhou, with identities ranging from 97.9% to 99.3%. The CRF68_01B sequence in this study exhibited high identity (98.9%) with a sequence reported in neighboring Anhui province. Eight URFs appeared to be closely linked to sequences from Beijing, Guizhou, Yunnan, Sichuan, Anhui and Hong Kong, with nucleotide identities ranging from 92.5% to 97.3%.

### Phylogenetic Analyses

**Figure 3:**
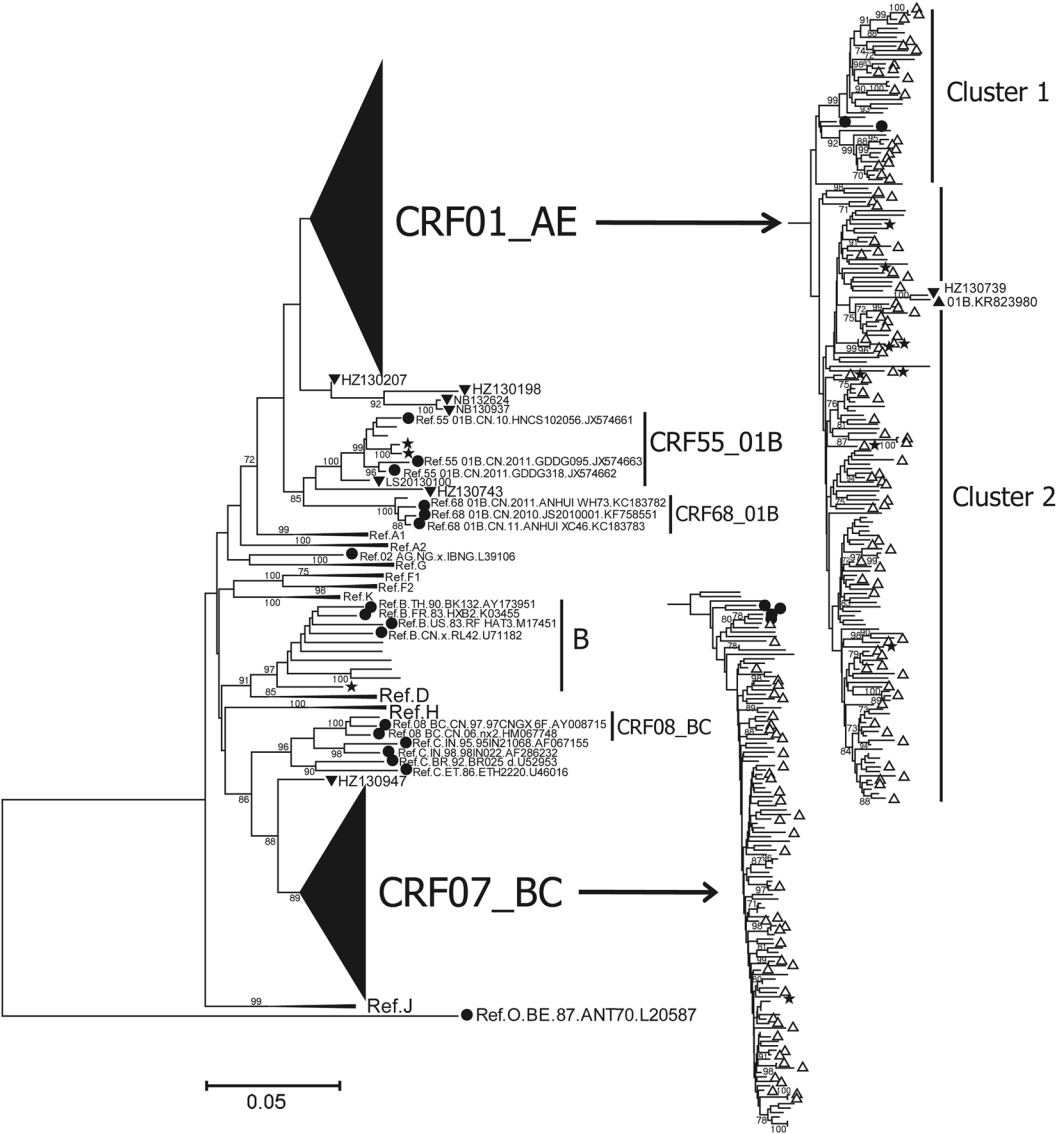
Neighbor-joining phylogenetic tree constructed based on partial *pol* genes from MSM who were newly diagnosed in 2013 in Zhejiang. The scale bar indicates 5% nucleotide sequence divergence. Values on the branches represent the percentage of 1000 bootstrap replicates, and bootstrap values over 70% are shown in the tree. The CRF01_AE cluster and the CRF07_BC cluster are compressed into a triangle on the left and expanded to show details on the right. The reference sequences obtained from the Los Alamos HIV database are marked as solid circles. The CRF01_AE reference sequences include Ref.01_AE.CN.05.05GX001.GU564221 and Ref.01_AE.TH.90.CM240.U54771. The CRF07_BC reference sequences include Ref.07_BC.CN.05.XJDC6431_2.EF368372, Ref.07_BC.CN.05.XJDC6441.EF368370, Ref.07_BC.CN.98.98CN009.AF286230 and Ref.07_BC.CN.97.97CN001.AF286226. Sequences with present address of Hangzhou are indicated by hollow upward-pointing triangle (△). URFs are labeled with solid downward-pointing triangles (▼), and their sample ID is indicated. The sequences with SDRMs are marked as five-pointed stars (★). The previously reported 01B strain with accession number KR823980 is labeled with a solid upward-pointing triangle (▲).

**Figure 4:**
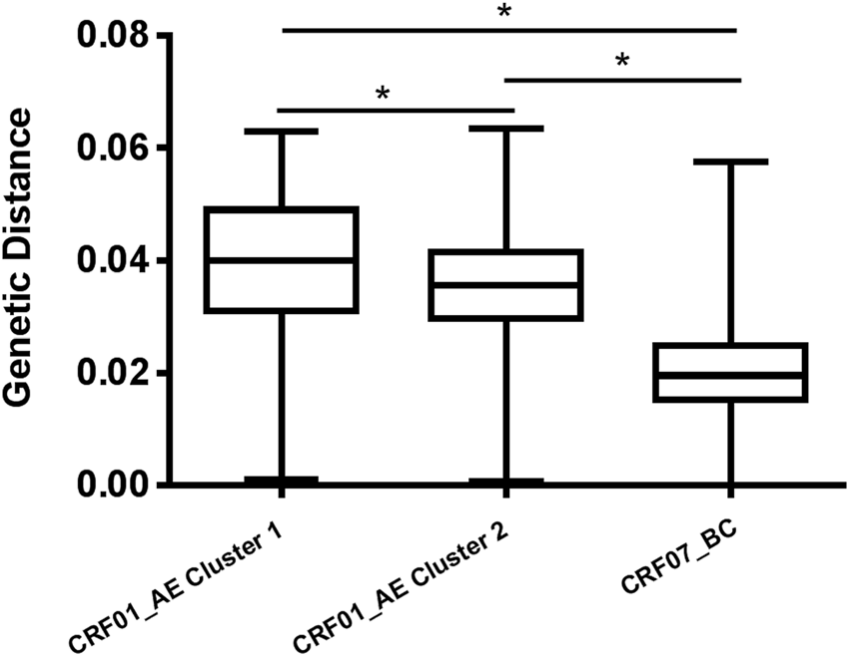
Nucleotide diversity of distinct HIV-1 variants among MSM in Zhejiang province. Nucleotide diversities of CRF01_AE (clusters 1 and 2) and CRF07_BC are shown in comparison with each other. An asterisk (*) indicates a statistically significant difference (*P* < 0.001) in nucleotide diversity between two compared categories.

One hundred seventy-four strains of CRF01_AE demonstrated an obvious clustering phenomenon, with two distinct CRF01_AE clusters (clusters 1 and 2) (Fig. 3). CRF01_AE cluster 2 could be divided into several sub-clusters. In contrast, CRF07_BC strains were scattered and did not gather in sub-clusters. The genetic distances within CRF01_AE cluster 1, cluster 2, and the CRF07_BC group were 0.039 ± 0.012, 0.035 ± 0.009, 0.021 ± 0.009, respectively (Fig. 4). The genetic distances between any two groups were significantly different (*P* < 0.001). No statistically significant differences were found in genetic distances between Hangzhou and the whole province within CRF01_AE cluster 1 (0.037 ± 0.014 vs 0.039 ± 0.012, *P* = 0.152) and CRF01_AE cluster 2 (0.035 ± 0.009 vs 0.035 ± 0.009, *P* = 0.757). Despite statistical differences (0.020 ± 0.007 vs 0.021 ± 0.009, *P* < 0.001), the difference of genetic distances between Hangzhou and the whole province in CRF07_BC group was far too small and actually meaningless.

According to Fig. 3, no clusters demonstrating geographical features were identified, suggesting a complex and intertwined network across the province. This indicates that the spread of the epidemic was not localized to a particular region but occurred within a whole province or potentially an even larger region than we had imagined. It was intriguing that the strains located in Hangzhou (inferred from the patients) diffused across the phylogenetic tree as a whole. That is, the strains with an imprint of Hangzhou were scattered throughout the entire province, indicating that the circulating strains among MSM in Zhejiang province possess a close-knit genetic relationship with Hangzhou.

### Transmission network

**Figure 5:**
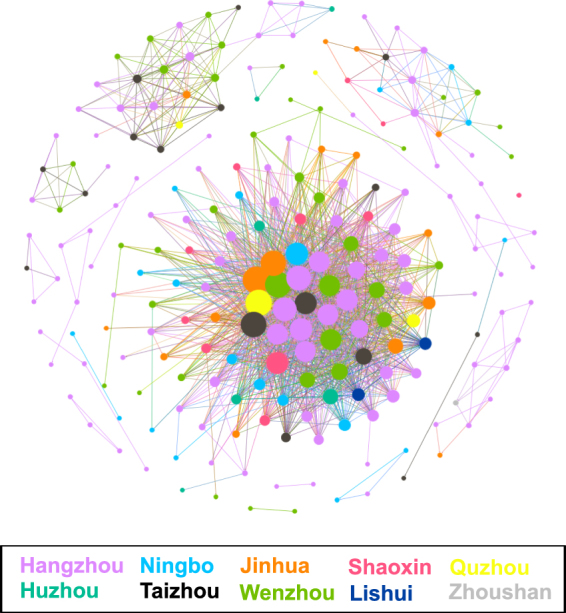
HIV-1 regional transmission network inferred from *pol* sequences. The network contains 214 nodes and 1798 edges. Edge lengths are optimized for visual presentation and do not represent genetic distances between each other. Node size is proportional to the numbers of its edges. Colors correspond to geographic regions.

An HIV-1 transmission network was constructed and is shown in Fig. 5. It contains 214 nodes and 1798 edges, with an average of 16.80 edges per node. Among the 1798 edges, 24.2% (435 edges) of edges connect two nodes that each represents a patient in the same region, with Hangzhou accounting for 72.0% (313/435). Edges that connect two nodes from different regions were in the majority, with a proportion of 75.8% (1363/1798), of which edges that connected Hangzhou to other regions accounted for 62.4% (850/1363). Patients in Hangzhou were estimated to have an average of 2.25 ± 4.73 potential local transmission linkages, while patients in other regions had an average of 0.71 ± 1.60. A significant difference was found between Hangzhou and other regions (Z = −3.941, *P* < 0.001). Similarly, a significant difference was found in potential cross-regional transmission linkages on average between Hangzhou and other regions (6.12 ± 11.36 vs 2.98 ± 6.75, Z = −2.116, *P* = 0.034). We determined that 64.7% (1163/1798) of edges connected at least one node representing a patient in Hangzhou. Up to 73.1% (850/1163) of edges connect one node from Hangzhou to another node from another region, inferring a potential transmission between Hangzhou and other regions. Hangzhou demonstrated apparent genetic relationships with all other studied regions, as inferred from the transmission network (Fig. 5). Moreover, Hangzhou had the largest number of nodes with ≥50 edges, accounting for 50.0% (10/20), indicating a remarkable role for the most sexually active individuals.

### Transmitted Drug Resistance

According to the CPR analysis performed on 311 sequences, 12 (3.9%, 12/311) sequences were identified as having SDRMs (Table 3), representing an overall low level of TDR. The proportions of sequences with drug resistance mutations (DRMs) to protease inhibitors (PIs), nucleoside reverse transcriptase inhibitors (NRTIs) and non-nucleoside reverse transcriptase inhibitors (NNRTIs) were 1.9% (6/311), 1.0% (3/311), and 1.9% (6/311), respectively. A total of 15 DRMs comprising 13 different types were found, including 6 PI DRMs with 5 types, 3 NRTI DRMs with 2 types and 6 NNRTI DRMs with different types. Among PI-related DRMs, M46I (n = 2), G73S (n = 1), L76V (n = 1), I85V (n = 1) and L90M (n = 1) were identified. Among NRTI-related DRMs, M184V, which causes high-level resistance to lamivudine (3TC) and emtricitabine (FTC), was found in two patients. T215S was found in one patient. Among NNRTI-related DRMs, K101E, V106M, Y181C, Y188L, Y188H and G190A were found in one patient each. A total of 83.3% (10/12) patients with SDRMs each harbored a single resistance mutation and were infected with CRF01_AE strains (n = 8) or CRF55_01B (n = 2). Additionally, one patient (NB130889) infected with CRF07_BC had two DRMs (L76V and K101E) that conferred resistance to PIs and NNRTIs. Another patient (JH131907) infected with subtype B had three DRMs (L90M, T215S and Y188L) conferring resistance to PIs, NRTIs and NNRTIs at different levels.

**Table 3:** Characteristics of Patients with SDRMs.

Sample ID	Age	Diagnosis date	CD4 count	Location of reported cases	Household registered	Present address	Occupation	Marital status	HIV subtypes	SDRM		
										PI	NRTI	NNRTI
HZ130063	22	2013/4/9	445	Hangzhou	Hunan	Hangzhou	Commercial service worker	Single	CRF01_AE	—	M184V	—
HZ130197	45	2013/5/14	432	Hangzhou	Anhui	Hangzhou	Worker	Divorced/widowed	CRF01_AE	—	M184V	—
HZ131396	23	2013/3/22	405	Hangzhou	Hangzhou	Hangzhou	Domestic worker and unemployed	Single	CRF01_AE	G73S	—	—
HZ130065	44	2013/4/17	50	Hangzhou	Hangzhou	Hangzhou	Other	Single	CRF01_AE	—	—	V106M
HZ131412	35	2013/5/22	78	Hangzhou	Hangzhou	Hangzhou	Cadre	Married	CRF01_AE	M46I	—	—
NB130889	50	2013/5/9	172	Ningbo	Ningbo	Ningbo	Peasant	Married	CRF07_BC	L76V	—	K101E
WZ130854	37	2013/5/30	527	Wenzhou	Jiangxi	Wenzhou	Worker	Divorced/widowed	CRF55_01B	I85V	—	—
WZ130115	29	2013/6/21	515	Wenzhou	Yunnan	Wenzhou	Commercial service worker	Single	CRF55_01B	M46I	—	—
JH131907	42	2013/8/15	200	Jinhua	Jinhua	Jinhua	Commercial service worker	Married	B	L90M	T215S	Y188L
JH132173	23	2013/9/26	545	Jinhua	Jinhua	Jinhua	Peasant	Single	CRF01_AE	—	—	Y188H
JH131909	29	2013/8/15	293	Jinhua	Jiangxi	Jinhua	Migrant worker	Married	CRF01_AE	—	—	G190A
SX130033	43	2013/1/22	424	Shaoxin	Jiangsu	Shaoxin	Domestic worker and unemployed	Divorced/widowed	CRF01_AE	—	—	Y181C

Of the 12 cases with SDRMs, 58.3% (7/12) were 35 years old and above, and 41.7% (5/12) resided in Hangzhou and were locally confirmed as HIV-positive. Workers and peasants were the most frequent occupations, and the predominant subtype was CRF01_AE (66.7%, 8/12). It was notable that 2 migrants living in Wenzhou were infected with CRF55_01B with PI DRMs.

## Discussion

In the present study, we conducted a systematic HIV molecular epidemiological survey among newly diagnosed HIV-infected MSM across Zhejiang province. Based on phylogenetic analyses of partial *pol* sequences, the HIV epidemic in MSM is characterized by multiple HIV-1 subtypes and emerging complex recombinants. However, it is still dominated by CRF01_AE and CRF07_BC. Cross-regional HIV transmission in MSM was common, inferred through phylogenetic analysis and the constructed transmission network, suggesting the existence of an interwoven complex network in which Hangzhou likely plays a central regional role in the intra-provincial spread of HIV. We also estimated a low prevalence of primary HIV DR among MSM in Zhejiang. The overall research results provided a comprehensive view of HIV-1 genetic characterization in MSM, which is conducive to a deeper understanding of HIV epidemiology and the implementation of evidence-based interventions among MSM in Zhejiang province.

Although the major HIV-1 subtypes identified were still CRF01_AE and CRF07_BC in MSM, which is similar to findings from other reports^7,8,12–14,21^, certain new CRFs and URFs have emerged, pointing to a continual increase in HIV genetic diversity. CRF55_01B was first identified in Chinese MSM in 2012^22^, and this subtype accounted for 9.2% (99/1072) of all subtyped sequences in a reported outbreak among MSM in Shenzhen^23^. Moreover, a recent paper reported that 19 strains of CRF55_01B were identified from 975 MSM in 7 provinces, with prevalences ranging from 1.5% to 12.5%^24^. Obviously, CRF55_01B has disseminated widely among MSM in China. It was not surprising that we identified 4 CRF55_01B strains with a prevalence of 1.3% in this cross-sectional study, and this result confirms that the national epidemic situation involves CRF55_01B. CRF68_01B was first identified in Anhui in 2013^13^, followed by Jiangsu^25^ and Shanghai^14^. When the known sporadic cases of CRF68_01B were summarized, they appeared to be relatively concentrated in southeast China, particularly the Yangtze River Delta region, one of China’s most prosperous regions, which implied that this subtype might originate there and would become widespread in the near future, similar to CRF55_01B. In addition to the newly designated CRFs, we identified 8 URFs, of which 4 were URF(CRF01_AE/B) and the remainder were URF(CRF01_AE /07_BC). Higher rates of dual-variant and multiple-variant HIV infection have been reported in MSM compared to heterosexual individuals in the same populations^6,26^, increasing the chances for HIV recombination. There is growing concern that URFs may be prone to formation in MSM due to the high-risk behavior features of this population, including multiple sex partners, low rates of condom use and anal intercourse. In light of the fact that CRF01_AE, B and CRF07_BC are currently co-circulating among MSM in China^4,7–14,19,21^, several studies have detected various recombinants and CRF candidates among MSM in different regions of China, most of which involved recombinations between CRF01_AE and subtype B (or CRF07_BC)^7,12–14,18,19,21,25,27^. We perceived that the findings in this study might epitomize the HIV variations present in MSM in China. Using HIV BLAST, we identified close-knit genetic relationships among the 8 URFs with other provinces in China, indicating that widespread existence of the formed URFs. Moreover, we found 3 epidemiologically unlinked sequences with similar mosaic patterns, which might be CRF candidates after analysis of full-length genome sequence. It is easy to foresee that URF recombinant events will continue to rise in frequency in the near future, necessitating the implementation of effective intervention strategies in Chinese MSM. It was intriguing that 61.5% (8/13) of patients infected with novel CRFs/URFs were married or divorced/widowed, implying bisexual behaviors. Although there were no statistically significant differences compared to patients infected with other strains (χ2 = 2.957, *P* = 0.085), further studies examining the relationship between bisexual behavior and higher infection risk for novel CRFs/URFs are warranted.

We found that the vast majority of strains could be divided into CRF01_AE cluster 1, cluster 2 and the CRF07_BC cluster, which was comparable to findings from other report^8^. As shown in Fig. 5, the genetic diversity of CRF07_BC (0.021 ± 0.009) is lower than that of CRF01_AE cluster 1 (0.039 ± 0.012) and cluster 2 (0.035 ± 0.009) (*P* < 0.001). The relatively higher level of nucleotide homogeneity in CRF07_BC suggests that it emerged later than CRF01_AE cluster 1 and cluster 2. Very recently, a study of phylogenetic dynamics among young MSM demonstrated that CRF07_BC drove a new wave of the HIV epidemic among Chinese MSM^7^. As shown in Fig. 3, CRF07_BC strains formed a number of phylogenetic transmission clusters with high bootstrap values (>70%), hinting at the rapid transmission. Additionally, we noted that CRF07_BC reached its highest proportion compared to our previous report^21^. CRF07_BC, which originated in 1993 in Yunnan province among injection drug users (IDUs)^28^, has dominated among IDUs (68.8%) and only represents a small proportion among MSM (0.8%) according to a report from the third nation-wide molecular epidemiological investigation in 2007^29^. Further study is required to explore the underlying reasons driving the rapid spread of the CRF07_BC strain among MSM.

Hangzhou, the capital and largest city of Zhejiang province, is the provincial center in terms of economy, culture, science, education and tourism. It is prosperous as a central city of the Yangtze River Delta Economic Region and is famous for its national history culture and world-renowned scenery travel. However, it accounted for the largest proportion (approximately 40%) of newly reported HIV/AIDS cases among MSM in Zhejiang province in 2013. Hangzhou not only reported the largest number of cases of HIV-infected MSM but also likely played a central role in the spread of HIV among MSM in Zhejiang province, as inferred from the phylogenetic analysis (Fig. 3) and the transmission network (Fig. 5) constructed in this study. It was intriguing that the majority of MSM strains from various prefectures demonstrated a close-knit genetic relationship with Hangzhou. Further analysis revealed that Hangzhou represented the dominant proportion, not only in terms of local transmission (72.0%) but also cross-regional HIV transmission (62.4%) based on the provincial transmission network (Fig. 5), highlighting its remarkable role in the intra-provincial spread of HIV. Additionally, of the cases infected with novel CRFs/URFs or strains with SDRMs, nearly half resided in Hangzhou and were locally confirmed as HIV-positive. It was difficult to understand these dynamics upon their initial identification. However, we found clues by integrating a literature review and surveillance data on MSM behaviors (data not shown). Due to the large influence of traditional culture, homosexuality remains highly stigmatized and discriminated against in China^4,30^. Chinese MSM populations are inclined to hide their sexual identity and engage in sex behaviors in other regions rather than staying in their hometown, where they may be easily recognized by acquaintances^4,12,30^. Most MSM concentrate into metropolises, where self-identity and sexual partners can easily be acquired^12^. Thus, a large social and sexual network has formed and expanded continually over time across the province or with a broader geographical scope, and within this network, HIV transmission occurs quietly and spreads. This study estimated that 75.8% of HIV transmission events were cross-regional, while only 24.2% of transmission events occurred locally, giving support to the inference described above. Based on this, we could infer that Hangzhou was likely the distribution center for HIV transmission among MSM during the early stage of the epidemic. The findings in this study are crucial for the development of targeted interventions for MSM in Hangzhou, which would be expected to exert an overall effect on the entire province.

Our survey revealed an estimated prevalence rate of drug-resistant HIV-1 strains of 3.9% among ART-naive MSM in Zhejiang, representing a low level of TDR, and this was compatible with our previous reports in Zhejiang province^21,27^, as well as the majority of reports in China concerning MSM^11,12,20^. Previous studies reported that relatively high rates of DRMs among Chinese ART-naive MSM were associated with PIs^11,20^, which is included in second-line ART regimens and has limited usage in China. It was estimated that the majority of PI-DR strains were derived from foreign countries with prolonged ART history. However, RTI SDRMs (9 cases) were more numerous than PI SDRMs (5 cases) in this study, similar to a previous report from Kunming^12^. An annual ART report showed that 85.4% of cases used first-line ART regimens (2 NRITs + 1 NNRTI), while only 14.6% used second-line regimens in Zhejiang province in 2013 (data unpublished). The mutations M46I, L90M, M184V, K101E G190A and Y181C, found in 8 cases (66.7%) in this study, were listed as the top five categorized DRMs in ART patients across the province. Given the 10-year implementation of HAART in Zhejiang, the higher prevalence of RTI mutations compared to PI mutations is likely attributable to the broader and longer use of first-line regimens. This indicates that DR strains were likely derived from domestic transmission, highlighting the importance of timely and effective intervention measures for TDR. However, we did not identify a predominant DRM, suggesting the presence of extensive sources for DR strains. Although it is unnecessary to make significant adjustments to current ART regimens, the prevalence of DR strains could compromise ART to a certain extent. Further studies are essential to trace the sources of DR strains and curb the epidemic.

In conclusion, our study provides clues regarding the characteristics of the HIV-1 epidemic among MSM in Zhejiang province and also sheds light on the national epidemic. The results of this study are conducive to the design and implementation of evidence-based interventions. Further study is still needed to elucidate the sources of novel CRFs/URFs and strains with SDRMs as well as the temporal and spatial migration patterns of HIV among different populations.

## Methods

### Ethics statement

This study was approved by the Ethical Review Committee of the Zhejiang Provincial Center for Disease Control and Prevention, and written informed consent was obtained from all patients involved in this study. All the methods in this study were carried out in accordance with the approved guidelines and regulations.

### Subjects and Sample Collection

A total of 340 newly diagnosed HIV-positive MSM cases, without antiviral therapy, were recruited between January and December 2013 across Zhejiang province (the geographical location is highlighted in Fig. 1). Ten out of eleven prefectures (except for Jiaxin) were covered in the study. The sample for the study was proportionally stratified to be representative according to case reports for each prefecture. The numbers of enrolled subjects were strictly proportional based on the reported cases in local regions as well as the fundamental demographic composition. The numbers of study subjects for each prefecture were as followed: Hangzhou (n = 143), Ningbo (n = 42), Wenzhou (n = 49), Huzhou (n = 12), Shaoxing (n = 20), Jinhua (n = 31), Quzhou (n = 7), Zhoushan (n = 4), Taizhou (n = 29) and Lishui (n = 3). HIV-1 infection status was confirmed by western blotting assay (HIV BLOT 2.2, MP Diagnostics, Singapore). Six to ten milliliters of EDTA anticoagulated blood was collected from the study patients within 1–3 months after their infection was confirmed. Plasma was separated within 6 hours after collection and frozen at −80 °C for further use. Patient demographic data were collected, including age, ethnicity, original registered residence, current residence, education status, marital status, occupation, and other information.

### RNA Extraction and Amplification

HIV RNA was extracted from 140 µl of plasma using a QIAamp Viral RNA Mini Kit (Qiagen, Germany) according to the manufacturer’s instructions. The RNA was utilized for reverse transcription PCR and nested PCR to generate *pol* fragments (HXB2: 2147–3462) according to procedures described previously^27^. The *pol* fragment was approximately 1316 bp in length and covered the entire protease (PR) gene and the first 300 codons of the reverse transcriptase (RT) gene. PCR amplification was carried out in a thermal cycler (Veriti, Applied Biosystems, Carlsbad, CA) and then analyzed by performing 1% agarose gel electrophoresis with the PCR products. Positive samples were sent to Beijing Zixi Bio Tech Co. for purification and sequencing using five overlapping primers in an ABI 3730XL sequencer (Applied Biosystems, Carlsbad, CA).

### Sequence Assembly and Phylogenetic Analysis

The sequences were trimmed, assembled and checked with Sequencher v5.0 (Genecodes, Ann Arbor, MI). ClustalW multiple alignment and manual editing were performed using BioEdit v7.2.0 (Ibis Biosciences, Carlsbad, CA). Reference sequences covering the major HIV-1 subtypes and CRFs were retrieved from the Los Alamos HIV sequence database (http://www.hiv.lanl.gov). MEGA v6.0 was used to construct a phylogenetic tree employing the Neighbor-joining method based on the Kimura two-parameter model with 1000 bootstrap replicates. Potential intersubtype recombinations were first analyzed using the Recombination Identification Program (RIP) v3.0, which integrates with the LANL HIV database (http://www.hiv.lanl.gov/), and then verified via bootscanning and informative site analysis using the program Simplot v3.5.1^31^. To determine the most probable geographic origin of novel HIV variants circulating in Zhejiang province, each novel subtype/URF sequence was aligned with HIV-1 sequences isolated worldwide with the highest identities using the online tool HIV BLAST, available at the LANL HIV database.

### Network Construction

Pairwise genetic distances were computed using MEGA v6.0, and only those pairs whose divergence was ≤1.5% (0.015 expected substitutions per site)^32^ were used for network analysis. The network was constructed by identifying pairs of sequences (nodes) and their potential transmission relationships (edge) using the visualization software Gephi 0.9.2.

### Genotypic Analysis of HIV-1 Drug Resistance

Eligible nucleotide sequences for the *pol* gene in FASTA format were submitted to the online Calibrated Population Resistance (CPR) Tool v6.0^33^ (http://cpr.stanford.edu/cpr.cgi), available at the HIV Drug Resistance Database (http://hivdb.stanford.edu). Nucleotide sequence sets were analyzed for proportions with Surveillance Drug Resistance Mutations (SDRMs) based on the list recommended by the WHO in 2009^34^.

### Statistical Analyses

Statistical analyses were carried out using Statistical Product and Service Solutions (SPSS) v19.0 (IBM, Armonk, NY). Differences between the subtype distributions of any two groups were assessed using Person’s χ^2^ test or Fisher’s exact test. Differences between the genetic distances of any two groups were assessed using the Independent sample t test. Differences between potential transmission linkages of any two groups were assessed performing the Wilcoxon rank-sum test. *P* values < 0.05 were considered statistically significant.
